# The relevance of restrained eating behavior for circadian eating patterns in adolescents

**DOI:** 10.1371/journal.pone.0197131

**Published:** 2018-05-23

**Authors:** Stefanie A. J. Koch, Ute Alexy, Tanja Diederichs, Anette E. Buyken, Sarah Roßbach

**Affiliations:** 1 DONALD Study, Nutritional Epidemiology, Department of Nutrition and Food Sciences, Faculty of Agriculture, University of Bonn, Bonn, North Rhine-Westphalia, Germany; 2 Public Health Nutrition, Institute of Nutrition, Consumption and Health, Faculty of Natural Sciences, University Paderborn, Paderborn, North Rhine-Westphalia, Germany; University of Lübeck, GERMANY

## Abstract

**Background:**

Restrained Eating, i.e. the tendency to restrict dietary intake to control body-weight, often emerges during adolescence and may result in changes in circadian eating patterns.

**Objective:**

The objective of the present investigation was to determine the cross-sectional relevance of restrained eating for characteristics of circadian eating pattern in adolescents and whether changes in restrained eating are accompanied by concurrent changes in circadian eating pattern over the course of adolescence.

**Methods:**

Two questionnaires assessing restrained eating (Score 0–30) with parallel 3-day weighed dietary records from two different time points were available from 209 (♂:101, ♀:108) 11–18 year old adolescents of the DONALD study. Mixed linear regression models were used to analyze whether restrained eating was associated with eating occasion frequency, snack frequency and morning and evening energy intake [in % of daily energy intake, %E]. Linear regression models were used to examine whether changes in restrained eating were associated with changes in the mentioned variables.

**Results:**

Among girls, greater restrained eating was cross-sectionally associated with higher morning energy intake (p = 0.03). Further, there was a tendency towards lower evening energy intake with higher levels of restrained eating for the whole sample (p = 0.06). No cross-sectional associations were found with eating occasion or snack frequency. Each one-point increase in restrained eating during adolescence was related to a concurrent decrease in eating occasion frequency by 0.04 (95% CI -0.08; -0.01, p = 0.02) and in evening energy intake by 0.36%E (95% CI -0.70; -0.03, p = 0.04). A tendency towards decreasing snack frequency with increasing restrained eating was observed (β = -0.03, 95% CI -0.07; 0.00, p = 0.07). No association was found between changes in restrained eating and concurrent changes in morning energy intake.

**Conclusion:**

We found indications for cross-sectional and prospective associations between restrained eating and chronobiological aspects of food intake in adolescents. Our results suggest that restrained eating should be considered a relevant determinant of circadian eating patterns.

## Introduction

Irregularities and alterations in circadian eating pattern (e. g. irregular meal times or preferred consumption of food in the evening/night) are thought to play a role in the development of type 2 diabetes mellitus, obesity and cardiovascular diseases [1–3]. Eating occasion frequency, snack frequency and daytime-specific energy intakes, e.g. morning or evening energy intake, are related to timing and rhythms of food intake. Therefore they can be considered as characteristics of circadian eating. Restrained eating behavior (RE) is described as the tendency to restrict dietary intake with the intention to lose body weight or avoid weight gain [4]. Hence this eating style is under cognitive rather than physiological control [4, 5]. Although RE is highly related to dieting, it is not equivalent, mainly because RE refers to the intent (not the action) to diet. Nevertheless, RE has often been associated with a restriction in total energy intake [6–10], but also with disinhibited overeating as a consequence of a loss of cognitive control [11, 12]. Restrained eaters exhibit an altered everyday eating behavior compared to non- or less-restrained eaters [13, 14], which is not only reflected by their food-choices [13, 14] and a lower total energy intake [6–10]: Lower meal and snack frequencies [14, 15] have also been observed in individuals with a higher level of RE and weight-control behavior in adolescents has been associated with a more common breakfast skipping [14–16]. Further, self-regulation is thought to be a resource that appears to decrease over the course of the day [17] and food intake late in the day is discussed to be less satiating than in the morning [18]. Therefore, it might appear that adolescents with a cognitive controlled eating behavior are more vulnerable to disinhibited overeating in the evening than earlier in the day. Techniques of restrained eaters to restrict their dietary intake therefore seem to be associated with characteristics of circadian eating pattern. Thus RE can be considered a potential determinant of circadian eating.

It appears that young adolescents are at particular risk for RE, as they are vulnerable for weight concerns, body dissatisfaction and problematic eating behaviors [19, 20]. Symptoms of eating disorders in adolescence are particularly more commonly found among girls than boys [21] and this gender-difference even extends to RE [15, 20, 22]. Adolescence is a developmental period of particular importance for shaping long-term eating habits and is considered a critical phase for the development of obesity and type 2 diabetes mellitus [23–25]. Several studies further observed higher BMI and other measures of obesity among persons with greater RE [26–30]. To elucidate whether RE determines aspects of circadian eating patterns during adolescence could contribute to the understanding of how RE potentially affects the development of chronic diseases like obesity in adulthood. We hypothesized, that adolescents with higher levels of RE would have less eating occasions and lower food intake in the morning but higher food intake in the evening, when cognitive resources for self-control are exhausted. Further, we hypothesized that changes in RE during adolescence are accompanied by concurrent changes in eating frequency and day time specific energy intake.

To our knowledge, no study has yet explicitly investigated the association between RE and circadian eating patterns among adolescents. Using data from the Dortmund Nutritional Anthropometric Longitudinally Designed (DONALD) study, we therefore evaluated cross-sectionally whether RE is associated with eating occasion frequency, snack frequency, morning energy intake and evening energy intake in German adolescents. Furthermore, we analyzed whether individual changes in RE during adolescence are associated with concurrent changes in these characteristics of circadian eating patterns.

## Methods

### Study population

The DONALD study is an ongoing open cohort study which collects information on diet, growth, development and metabolism of healthy participants between infancy and adulthood. Since 1985, approximately 30–35 infants are newly recruited every year and first examined at the age of 3 months. Each child is scheduled to return for 3 more visits in the first year, two in the second, and annually thereafter until young adulthood. The DONALD study is exclusively observational and non-invasive until the age of 18 years. Regular examinations include 3-day weighed dietary records, anthropometric measurements and interviews on lifestyle. Further details on the DONALD study design were published previously [31]. The study was approved by the Ethics Committee of the University of Bonn and all examinations are performed with parental and later on participants written consent.

In the DONALD study, RE has been assessed since 2005 in participants between 11 and 18 years. These assessments are scheduled for visits at 11, 14 and 18 years of age. If a participant fails to attend these visits, he/she is interviewed at the next visit. Through May 2016, a total of 886 questionnaires on RE were collected from 494 participants. Questionnaires with more than one missing answer were excluded from the analyses (n = 11). For 658 questionnaires (from 392 participants), dietary records collected at the same visit were available. Overall, 218 participants provided at least two RE questionnaires with parallel 3-day weighed dietary records. For participants, who had provided more than two questionnaires and dietary records, the first and the last available RE questionnaire and dietary record were used to assess changes in RE and eating pattern. Two-hundred and nine participants additionally provided the relevant data to calculate the age at the onset of the pubertal growth spurt (age at take-off), a marker for initialization of puberty [32] and a potential confounder. Therefore, the final sample consisted of 209 participants (♀: 108, ♂: 101) providing 418 questionnaires and 3-day weighed dietary records.

### Restrained eating behavior

RE was assessed using the Dutch Eating Behavior Questionnaire for children (DEBQ-K) by Franzen and Florin [33]. This questionnaire is an age adapted German version [34] of the Dutch Eating Behavior Questionnaire developed by van Strien et al. [35] and has been validated for the assessment of RE in German-speaking children and adolescents [33, 36, 37]. In the DONALD study, a subscale of the DEBQ-K was used to assess RE, with 10 items and four potential responses (never, seldom, sometimes and often). After an introduction by the study staff, the questionnaire was answered by the participants in a separate room without the presence of the parents. Each response was scored ranging from 0 (never) to 3 (often). Subsequently, an individual overall RE-score was calculated by summing the scores for each item. The final individual scores could range from 0 to 30, with a higher score indicating higher RE. In case of a missing answer, the individual median was used to replace the missing value (n = 2).

### Dietary assessment

Dietary intake in the DONALD study is assessed by use of 3-day weighed dietary records. All foods and beverages consumed, as well as leftovers, are weighed and recorded over 3 consecutive days by the participants with the use of regularly calibrated electronic food scales [initially Soehnle Digita 8000 (Leifheit AG, Nassau, Germany), now WEDO digi 2000 (Werner Dorsch GmbH, Muenster/Dieburg, Germany)]. When exact weighing is not possible, household measures (e.g. spoons, cups) are allowed for semi-quantitative recording [31]. Information on recipes and on the types and brands of food items consumed is also requested. Additionally, participants record the daytime of every eating occasion.

Energy intakes were calculated using the continuously updated in-house nutrient database LEBTAB [38], which is based predominantly on German and US food composition tables. Energy and nutrient contents of commercial food products are calculated by recipe simulation using labelled nutrient contents and ingredients. Total energy intake was calculated as the individual mean of total energy intake on the three days of recording. Total energy intake is an important indicator of the construct validity of RE and thus for the interpretation of the present results and was considered as an additional outcome variable in the cross-sectional analyses.

### Definitions of circadian eating pattern

#### Variables of eating frequency

We chose to define variables of eating frequency by determining time- and energy-based cut-offs. This approach is independent of individuals perceptions of what constitutes a meal/snack and has already been used in previous evaluations of DONALD data [39, 40], which makes findings more comparable over time and across studies [39–41]. Accordingly, all foods and beverages consumed within a 30 min time period were summarized into one eating occasion, and all eating occasions <10 kcal were added to the previous eating occasion. Eating occasions providing ≤10% of total energy intake were defined as snacks. Subsequently, the individual means of the daily eating occasion frequency and snack frequency were calculated from the three record days.

#### Morning and evening energy intake

Morning energy intake was defined as all dietary intakes between the age-specific end of the night and 11am and evening energy intake was defined as all dietary intakes between 6pm and the age-specific beginning of the night. This procedure has been used in previous evaluations of DONALD data [42] and derives from preliminary analysis, showing that children and adolescents (2–18 years) of the DONALD study consume their first (and second) breakfast until 11 a.m., and that 6 p.m. marks the point in time between afternoon snacks and the main evening meals. The age-specific beginning and end of the night was estimated by using all available complete 3-day dietary records from DONALD participants of the respective age. The age specific beginning of the night was defined as the time of the day, after which less than 5% of the last eating occasions (≥10 kcal) before midnight were documented. The age-specific end of the night was the time of the day past 5 a.m., after which more than 5% of the first eating occasions (≥10 kcal) were documented. Energy intakes in the morning and in the evening were considered as percentage of total energy intake, respectively. Subsequently, the individual means of daily morning and evening energy intake were calculated from the three record days.

### Assessment of potentially confounding factors

The following variables were considered as potentially confounding factors and tested for their statistical relevance: age (years), sex (male/female), body mass index standard-deviation-score (BMI-SDS), age at take-off (years), duration of breast feeding (≥4 months fully breast fed: yes/no), maternal overweight (BMI≥25kg/m^2^/BMI<25kg/m^2^), maternal educational status (≥12 years of schooling: yes/no) and maternal employment (yes/no), number of weekdays per 3-day dietary record (1/2/3).

Anthropometric measurements are done at the annual visit at the DONALD study center. Participant’s height and weight were measured according to standard procedures with the participants dressed in underwear only and barefoot. Standing height was measured to the nearest 0.1cm using a digital stadiometer (Harpenden). Body weight was measured to the nearest 100g using an electronic scale (Seca 753E; Seca Weighing and Measuring System). BMI was calculated as the body weight (kg) divided by the square of the body height (m). Sex- and age-dependent BMI-SDS were calculated on basis of reference values for German children and adolescents by Kromeyer-Hauschild et al. [43]. To define overweight, obesity and underweight, the cut off-value 90^th^ percentile, 97^th^ percentile and 10^th^ percentile was used, respectively [43].

Age at take-off, an early pubertal marker, was defined as the age at minimal height velocity at the onset of the pubertal growth spurt [32]. It has been calculated by the use of individual height measurements of the participants. Further details on its calculation were described elsewhere [32]. Age at take-off has been considered a potentially confounding factor so as to adjust for individual differences attributable to pubertal development. Specifically, the onset of puberty marks the beginning of a lifespan known to considerably affect eating patterns [39, 44, 45]–which may extend to circadian eating pattern (i.e. the outcome of the present analysis). In addition puberty is also characterized by a high risk for RE [6, 19, 46, 47], hence puberty onset is potentially associated with our predictor.

The duration of breast-feeding was inquired with a standardized questionnaire. At each of the first visits at 3, 6, 9, 12 and 18 months mothers were asked if they still breastfeed their infant, until the infant was fully weaned. For missing values the respective median of the total sample was used (n = 2). The duration of breast feeding has been considered a potentially confounding factor, because it has been associated with children’s eating behaviors [48] and with greater appetite regulation and self-control in later childhood [49, 50].

Parental body weight and height were measured with the same equipment as for the study participants. Parental overweight was defined as a BMI ≥25kg/m^2^ and has been considered a potentially confounding factor because parental dieting behavior and RE have been found to be transmitted in families from parents to their children [51–53]. High maternal educational status and maternal employment were inquired with a standardized questionnaire on their child’s admission to the study and at regular intervals thereafter. Socioeconomic disadvantages have been shown to be associated with dietary patterns in children [54] and with the development of RE in preadolescence [55].

### Statistical analysis

All statistical analyses were performed using SAS procedures (version 9.2; Cary, NC, USA). The significance level was set at a p-value of <0.05. A p-value <0.1 was interpreted as marginally significant and referred to as a tendency. As a former analysis of DONALD study data showed no difference in RE between adolescent boys and girls [46], sex-stratified analyses were performed in cases of significant sex interactions only. Descriptive data are presented as medians with their interquartile range or frequencies and percentages.

#### Cross-sectional-analysis

In cross-sectional analyses, per participant data from two different time points of 209 DONALD participants were used, resulting in 418 included measurements. To analyze the cross-sectional association between RE and characteristics of circadian eating patterns as well as total energy intake, linear mixed-effects regression models, including both fixed and random effects, were used (PROC MIXED in SAS). RE-score (0–30) was the principal fixed effect. The following variables were considered as outcome variables in separate models: eating occasion frequency, snack frequency, morning energy intake, evening energy intake and total energy intake. A repeated statement was considered to account for the lack of independence between repeated measures from the same person. Random effects were considered to allow variation between individuals and families with respect to the initial level (intercept) as well as linear, quadratic and cubic age trends of the respective outcome. The Akaike Information Criterion was used for model selection. Results are presented in tertiles of RE-score.

#### Change-on-change-analysis

For change-on-change analyses, individual changes in RE and dietary outcomes of the 209 participants were calculated subtracting baseline from endpoint values, resulting in 209 included observations. Whether individual changes in RE during adolescence are associated with concurrent changes in circadian eating pattern was examined by use of linear regression models (PROC GLM in SAS). Individual changes in RE were obtained by subtracting the RE-score at baseline from the RE-score at endpoint (change in RE = score at endpoint−score at baseline, range: -30 to +30). The change in RE-score was considered as fixed effect. Outcome variables were changes in eating occasion frequency, snack frequency, morning energy intake and evening energy intake, calculated by subtraction of the respective baseline from endpoint values. Results are presented as regression coefficients.

#### Adjustments

Basic models (model A) of cross-sectional analyses were adjusted for age. Basic models of change-on-change-analyses were adjusted for age at baseline, the time-span (in years) between baseline and endpoint and the RE-score at baseline. Subsequently, other relevant confounding factors were considered as covariates.

For the change-on-change analyses relevant categorical confounders were used from baseline (maternal educational status, maternal employment); relevant continuous variables were included by subtracting baseline from endpoint values (BMI-SDS).

Variables that significantly modified regression coefficients in the basic models by ≥10% or had a significant, independent effect on the outcome variable were considered relevant and were subsequently included in the final models [56, 57]. For cross-sectional analyses, variables leading to an improvement of the Akaike Information Criterion by more than 2 points were also considered relevant and included in the final models [39, 58]. To ensure comparability, the same adjustment was used for all models. This adjustment was derived from the strongest predictor-outcome association (model B). Sensitivity analyses using individually constructed models for each outcome yielded similar results (data not shown).

#### Additional analyses

To test whether associations between RE and morning energy intake or evening energy intake were driven or affected by meal skipping—i.e. whether lower morning energy intakes were due to skipping breakfast and not having a smaller breakfast or whether lower evening energy intakes were due to skipping evening meals accordingly—additional analyses were conducted. Therefore, in additional analyses only food-records with energy intake in the morning or in the evening on all three days of food-recording were included (model C), respectively. Thus, all dietary records without any energy intake in the morning or in the evening on at least one day of recording were excluded. Accordingly, in cross-sectional analyses, 49 dietary records from boys and 43 dietary records from girls were excluded because no energy intake in the morning was recorded on at least one day of food recording. Furthermore, 50 dietary records were excluded from cross-sectional analyses because no energy intake in the evening was recorded on at least one day of food recording. In change-on-change-analyses 79 and 41 dietary records with no energy intake in the morning or in the evening on at least one day of food recording were excluded, respectively.

## Results

### Characteristics

Characteristics of the study population stratified by tertiles of RE are presented in Table 1, separately for girls and boys. Data on dietary characteristics of the study population are presented in Table 2 analogously. The ratio of measurements from boys and girls was nearly balanced (♀: 108, ♂: 101). The mean age at baseline was 12.6/12.4 years and the mean age at endpoint was 16.7/16.4 years for boys/girls (data in S1 Table). Due to the fact that data from the first and the last assessment were used to calculate changes in RE and eating pattern when more than two assessments were available, the majority of participants were 11 years old at baseline (♂♀: 57%) and 18 years old at endpoint (♂: 63%, ♀:58%) (data in S1 Table). The median score for RE including all measurements was 5 (range: 0–24) (data in S2 Table). Overall, female participants had higher scores in RE than male participants (maximum boys: 20; maximum girls: 24). None of the participants reached the highest possible RE-score of 30. 12% of the participants and 41% of participants’ mothers had overweight or obesity. In girls and boys, the highest proportion of overweight was found in tertile 3 of RE and the lowest in tertile 1, which is also reflected in differences in BMI-SDS. In contrast, the highest total energy intake was found in tertile 1 of RE and the lowest in tertile 3 for girls and boys, which is shown in the dietary characteristics in Table 2. Similarly, the highest values for evening energy intake were found in tertile 1 of RE and the lowest in tertile 3. Around 22% and 12% of the whole sample had no energy intake on at least one day of food-recording in the morning and in the evening, respectively. Participants recorded on average 5 eating occasions per day, of which one was categorized as a snack. The sample was characterized by a high parental educational level with 72% of participants’ mothers attending school for at least 12 years.

**Table 1:** Characteristics of DONALD-study participants (n = 209) stratified by tertiles of restrained eating behavior (RE) presented separately for boys (n = 101) and girls (n = 108).

	Tertile 1		Tertile 2		Tertile 3	
	♂	♀	♂	♀	♂	♀
Participant‘s characteristics						
n (questionnaires/dietary records)	73	73	67	74	62	69
n (participants)	56	53	53	57	44	46
Age [years]	14 (11; 18)	14 (11; 15)	14 (13; 18)	14 (12; 18)	14 (11; 17)	14 (11; 18)
RE Score [0–30]	0 (0; 1)	0 (0; 2)	5 (4; 7)	6 (5; 8)	13.5 (11; 16)	15 (12; 19)
Minimum	0	0	3	3	9	10
Maximum	2	2	8	9	20	24
ATO [years]	11 (10; 11)	9 (8; 9)	10 (10; 11)	9 (8; 9)	11 (10; 11)	9 (8; 10)
BMI-SDS	-0.23 (-0.96; 0.39)	-0.46 (-1.12; 0.21)	0.00 (-0.77; 0.58)	0.09 (-0.43; 0.52)	0.78 (0.41; 1.66)	0.83 (0.28; 1.20)
Body weight status ^a^						
Normal weight [n (%)]	58 (80)	57 (78)	55 (82)	68 (92)	38 (61)	53 (77)
Underweight [n (%)]	11 (15)	15 (21)	6 (9)	3 (4)	2 (3)	1 (1)
Overweight [n (%)]	3 (4)	1 (1)	6 (9)	2 (3)	13 (21)	8 (12)
Obesity [n (%)]	1 (1)	-	-	1 (1)	9 (15)	7 (10)
Breast-fed ≥ 4 months [n (%)]	50 (69)	48 (66)	43 (64)	51 (69)	43 (69)	47 (68)
Parental characteristics						
Maternal overweight ^b^ [n (%)]	28 (38)	28 (38)	25 (37)	31 (42)	23 (37)	35 (51)
High maternal educational status ^c^ [n (%)]	47 (64)	54 (74)	50 (75)	54 (73)	41 (66)	53 (77)
Maternal employment [n (%)]	53 (73)	59 (81)	53 (79)	57 (77)	53 (86)	54 (78)

Presented values are medians (25^th^; 75^th^ percentile) or frequencies (%) and are based upon 418 questionnaires on restrained eating and parallel 3-day-weighed dietary records of 209 DONALD-study participants

Abbreviations: ATO ≙ Age at Take-Off, BMI ≙ Body Mass Index, SDS ≙ Standard Deviation Score, %E ≙ percentage of total energy intake

^a^ Overweight: >90th percentile/Obesity: >97th percentile/Underweight: <10th percentile of BMI-SDS based on German reference curves [43],

^b^ BMI ≥25,

^c^ ≥12 years of schooling

**Table 2:** Dietary characteristics of DONALD-study participants (n = 209) stratified by tertiles of restrained eating behavior (RE) presented separately for boys (n = 101) and girls (n = 108).

	Tertile 1		Tertile 2		Tertile 3	
	♂	♀	♂	♀	♂	♀
Number of recorded weekdays						
1 [n (%)]	31 (42)	29 (40)	19 (28)	24 (32)	25 (40)	21 (30)
2 [n (%)]	19 (26)	13 (18)	11 (17)	17 (23)	13 (21)	12 (17)
3 [n (%)]	23 (32)	31 (42)	37 (55)	33 (45)	24 (39)	36 (52)
Total energy intake [kcal]	2270 (1985; 2653)	1809 (1561; 1982)	2166 (1912; 2543)	1712 (1483; 1991)	2059 (1748; 2688)	1604 (1388; 1998)
Morning energy intake [%E]	22.1 (14.6; 29.7)	25.6 (19.6; 30.3)	24.1 (18.8; 33.2)	25.5 (18.0; 32.2)	23.2 (17.7; 28.3)	25.8 (20.3; 34.4)
No energy intake in the morning						
Never [n (%)]	54 (74)	61 (84)	54 (81)	55 (74)	45 (72)	57 (83)
On 1 day [n (%)]	8 (11)	9 (12)	8 (12)	13 (18)	13 (21)	8 (11)
On 2 days [n (%)]	6 (8)	2 (3)	4 (6)	4 (5)	3 (5)	4 (6)
On 3 days [n (%)]	5 (7)	1 (1)	1 (1)	2 (3)	1 (2)	-
Evening energy intake [%E]	33.0 (28.0; 39.3)	29.4 (24.1; 34.5)	31.0 (23.7; 39.8)	27.6 (20.8; 37.0)	30.6 (26.3; 35.8)	26.5 (20.9; 35.1)
No energy intake in the evening						
Never [n (%)]	66 (90)	62 (85)	63 (94)	60 (81)	57 (92)	60 (87)
On 1 day [n (%)]	7 (10)	7 (10)	4 (6)	9 (12)	5 (8)	8 (12)
On 2 days [n (%)]	-	4 (5)	-	5 (7)	-	1 (1)
On 3 days [n (%)]	-	-	-	-	-	-
Eating occasion frequency [n/day]	5.3 (4.7; 6.3)	5.3 (4.7; 6.0)	5.3 (4.7; 6.0)	5.2 (4.7; 5.7)	5.3 (4.7; 6.3)	5.0 (4.0; 5.7)
Snack frequency [n/day]	1.3 (0.7; 2.0)	1.3 (0.7; 2.0)	1.7 (0.7; 2.3)	1.3 (0.7; 2.0)	1.3 (1.0; 2.3)	1.3 (0.7; 1.7)

Presented values are medians (25^th^; 75^th^ percentile) or frequencies (%) and are based upon 418 questionnaires on restrained eating and parallel 3-day-weighed dietary records of 209 DONALD-study participants

Abbreviations: %E ≙ percentage of total energy intake

### Cross-sectional analysis

Results of the cross-sectional analyses are shown in Table 3. Analyses showed an inverse association between RE-score and total energy intake (model B: p<0.0001) for the total sample. In girls, higher RE-scores were associated with higher morning energy intake (model B: p = 0.03). In boys, no association was observed between RE and morning energy intake. Upon exclusion of all food-records with no energy intake in the morning on at least one day of food-recording (model C) the association between RE and morning energy intake did not remain statistically significant, neither for girls (p = 0.15) nor for boys (p = 0.17). RE was not associated with evening energy intake. However, upon exclusion of all food-records with no energy intake in the evening on at least one day of food-recording (model C) a tendency towards a lower evening energy intake with higher RE-scores was seen for the whole sample (model C: p = 0.06). There was no cross-sectional association between RE and eating occasion frequency or snack frequency.

**Table 3:** Characteristics of circadian eating patterns stratified by tertiles of restrained eating behavior—Results of cross-sectional analyses (n = 418 measurements from 209 participants).

	Restrained Eating			p
	Tertile 1	Tertile 2	Tertile 3	
**Total energy intake (kcal)**^a^				
Model A	2012 (1926; 2101)	1957 (1870; 2048)	1813 (1734; 1897)	<0.0001
Model B	2025 (1937; 2118)	1963 (1875; 2055)	1797 (1713; 1885)	<0.0001
**Eating occasion frequency (n/day)**^a^				
Model A	5.3 (5.1; 5.5)	5.4 (5.2; 5.6)	5.2 (5.0; 5.4)	0.07
Model B	5.3 (5.1; 5.5)	5.4 (5.2; 5.6)	5.2 (5.0; 5.4)	0.20
**Snack frequency (n/day)**^a^				
Model A	1.4 (1.2; 1.6)	1.5 (1.3; 1.7)	1.4 (1.2; 1.6)	0.49
Model B	1.4 (1.2; 1.6)	1.5 (1.3; 1.7)	1.4 (1.2; 1.6)	0.55
**Morning energy intake (%E)**^a^				
Boys				
Model A	22.5 (20.1; 24.9)	25.0 (22.5; 27.6)	22.3 (19.7; 25.0)	0.34
Model B	22.6 (20.1; 25.1)	25.3 (22.7; 27.8)	21.8 (19.0; 24.6)	0.19
Model C^b^	26.5 (24.1; 28.8)	26.9 (24.3; 29.5)	25.6 (23.1; 28.2	0.15
Girls				
Model A	25.1 (22.7; 27.4)	26.5 (24.2; 28.6)	27.8 (25.5; 30.0)	0.01
Model B	25.2 (22.6; 27.6)	26.6 (24.3; 28.7)	27.7 (25.2; 30.1)	0.03
Model C^c^	27.9 (25.5; 30.1)	29.8 (27.7; 31.9)	29.2 (26.8; 31.5)	0.17
**Evening energy intake (%E)**^a^				
Model A	31.3 (29.6; 33.1)	30.2 (28.4; 32.1)	29.8 (27.9; 31.6)	0.36
Model B	31.7 (29.9; 33.5)	30.3 (28.4; 32.1)	29.3 (27.4; 31.3)	0.12
Model C^d^	33.2 (31.5; 35.0)	32.2 (30.5; 34.0)	30.7 (28.9; 32.6)	0.06

Presented values are least squares means (95% confidence interval) and are based upon 418 questionnaires on restrained eating and parallel 3-day-weighed dietary records of 209 DONALD-study participants

Abbreviations: %E ≙ percentage of total energy intake

^a^ Model A: adjusted for age. Model B: adjustment like in model A plus BMI-SDS, maternal educational status, maternal employment.

^b^ Additional analysis: adjustment like in model B, n = 153 questionnaires with parallel dietary records

^c^ Additional analysis: adjustment like in model B, n = 173 questionnaires with parallel dietary records

^d^ Additional analysis: adjustment like in model B, n = 368 questionnaires with parallel dietary records

### Change-on-change analysis

Results of the change-on-change analyses are shown in Table 4. The average time between assessments at baseline and endpoint was 4.0 years [SD: 1.7]. An increase in the RE-score by 10 units during adolescence was associated with 0.4 fewer eating occasions per day (model B: p = 0.02). Further, increases in RE during adolescence were related to a tendency for a decrease in both snack frequency (model B: β = -0.03, p = 0.07) and evening energy intake (model B: β = -0.3, p = 0.06). Upon exclusion of all food-records with no energy intake in the evening on at least one day of food-recording, an increase in the RE-score of 10 units was associated with a decrease in evening energy intake by 3.6%E (model C: p = 0.04). No association was observed between changes in RE during adolescence and concurrent changes in morning energy intake.

**Table 4:** Associations between changes (Δ) in restrained eating behavior and changes in characteristics of circadian eating patterns (mean time span of 4 years)—Results of change-on-change-analyses (n = 209).

	Δ Restrained Eating	
	β (95% CI)	p
**Δ Eating occasion frequency (n/day)**^a^		
Model A	-0.04 (-0.07; -0.00)	0.03
Model B	-0.04 (-0.08; -0.01)	0.02
**Δ Snack frequency (n/day)**^a^		
Model A	-0.03 (-0.07; 0.00)	0.09
Model B	-0.03 (-0.07; 0.00)	0.07
**Δ Morning energy intake (%E)**^a^		
Model A	-0.04 (-0.38; 0.29)	0.80
Model B	-0.02 (-0.37; 0.33)	0.91
Model C^b^	-0.18 (-0.56; 0.21)	0.36
**Δ Evening energy intake (%E)**^a^		
Model A	-0.32 (-0.65; -0.00)	0.05
Model B	-0.33 (-0.66; 0.01)	0.06
Model C^c^	-0.36 (-0.70; -0.03)	0.04

Values are based upon data of 209 DONALD-study participants providing questionnaires on RE with parallel 3-day dietary records for two different time points (Baseline and Endpoint) to calculate changes (Δ) by subtracting endpoint from baseline values.

Abbreviations: %E ≙ percentage of total energy intake

^a^ Model A: adjusted for age at baseline, time between baseline and endpoint, restrained eating score at baseline. Model B: adjustment like in model A plus change in BMI-SDS, maternal educational status at baseline and maternal employment at baseline.

^b^ Additional analysis: adjustment like in model B, n = 130 study participants

^c^ Additional analysis: adjustment like in model B, n = 168 study participants

## Discussion

The present study provides epidemiological evidence for a relevance of RE for characteristics of circadian eating pattern in a convenient sample of adolescents from the DONALD study. Our results suggest that adolescents with higher levels of RE differ from adolescents with lower levels of RE in their characteristics of circadian eating pattern. Further, changes in the level of RE during adolescence may result in changes in some characteristics of circadian eating pattern. It has been hypothesized that adolescents with higher levels of RE have less frequent eating occasions and show lower food intake in the morning and higher food intake in the evening. In contrast we found that higher levels of RE were associated with a higher energy intake in the morning in girls and with a lower energy intake in the evening in the total study sample. In line with our hypothesis, we found that decreasing levels of RE during adolescence were accompanied by a concurrent decrease in eating occasion frequency but also with a concurrent decrease in evening energy intake. Hence, we propose that the development of RE during adolescence affects chronobiological aspects of food-intake.

### Restrained eating and total energy intake

Total energy intake was considered an additional outcome in cross-sectional analyses because it is an important indicator of the construct validity of RE as assessed in this study. Basically, RE only reflects the intent (not the action) to restrict dietary intake and according to the restraint theory, restrained eaters are further vulnerable to disinhibited eating [11]. Therefore, scores on measurement scales for RE do not necessarily reflect an actual restriction of dietary intake. However, the terms dieting and dietary restraint are often used interchangeably and a number of observational studies [6–10] have confirmed an inverse association between RE and total energy intake. Consistent with these results, adolescents with higher levels of RE had significantly lower total energy intake in the present sample. This could be attributable to the use of the DEBQ-K for the assessment of RE since Laessle et al. found that the commonly used measurement scales for RE appear to measure different components of the restraint construct [59]. The subscales for dietary restraint of the DEBQ (on which the DEBQ-K [33] used in our study is based) and the Three Factor Eating Questionnaire appear to assess the successful dieting behavior component of restrained eating, which is represented by an actual restriction in energy intake. The Restraint Scale by Herman and Polivy [11] in turn appears to identify dieters regardless of their success in energy restriction. We thus conclude that in the present study adolescents exhibiting a more restrained eating behavior successfully restrict their dietary intake and consequently have a lower total energy intake. However, the level of RE was notably lower in the examined DONALD sample compared to the level reported from other examinations applying the DEBQ-K in healthy children [36, 60].

Nevertheless, it should be considered that adolescents with higher levels of RE might only report a lower total energy intake without actually eating less. RE is hence potentially associated with underreporting [46, 61], however there was no possibility to distinguish restrained eaters who under-reported their energy intake from restrained eaters with reduced energy intake on basis of the collected nutritional data [62].

### Restrained eating and eating occasion/snack frequency

The results from our cross-sectional analyses suggest that adolescents exhibiting higher levels of RE do not differ from those with lower levels of RE concerning their eating occasion frequency or snack frequency. In line with these findings, studies with adult participants consistently concluded that restrained eaters restrict their dietary intake by reducing the meal size (and hence its energy content) rather than by omission of eating occasions [8–10]. One of these studies even reported that those with a higher level of RE had a slightly higher meal frequency [10].

It should be noted that studies on RE among adolescents show a different picture [14, 15]: In a sample of 402 twelve-year-olds, highly restrained adolescents ate fewer meals and snacks than non-restrained [15]. Additionally, in 1019 adolescent participants (11–16 years) an inverse correlation between RE and snack frequency was found [14]. In concordance with these findings, in the present study an increase in RE during adolescence was associated with a decrease in eating occasion frequency and a tendency towards decreasing snack frequency was observed in the change-on-change analyses. More restrained adolescents therefore did not differ from less restrained adolescents in their frequency of eating occasions and snacks in the present sample, but individual changes in RE during adolescence seem to result in changes in these variables. Our prospective results therefore support the idea that adolescents who develop a more restrained eating behavior during adolescence save calories by eating fewer meals or snacks.

However the overall evidence base for this association is weak as the majority of results stem from secondary analyses of studies not focused on RE. Additionally prospective associations between changes in RE over the course of adolescence and concurrent changes in eating occasion frequency or snack frequency have not been investigated in other studies up to now. Therefore our results should be interpreted with caution and need further confirmation.

One potential explanation for the inconsistency among the few existing studies may be the use of different definitions for eating occasions ranging from time of day or food-based classifications to eating occasions identified by participants themselves, according to time-intervals in-between or energy content [41]. The choice of criteria affects the characterization of eating patterns and the great variety of definitions impedes a comparison of results across studies [63]. Due to the above mentioned differences in the assessment of restrained eating behavior, another impediment for the comparison of study results is the fact that in the existing studies different measurement scales for RE were used [59, 64].

### Restrained eating and morning/evening energy intake

The fact that higher scores of RE were cross-sectionally associated with higher morning energy intake in girls might largely be driven by less breakfast skipping in girls with higher levels of RE, since in the additional analyses (model C, excluding food-records with no energy intake in the morning on at least one day of food-recording), this association was not confirmed. Further, among those who do eat in the evening (model C), higher restrained adolescents had lower evening energy intake than their less or non-restrained peers. In prospective change-on-change-analysis, increasing RE during adolescence was associated with decreasing evening energy intake in those who did eat in the evening (model C), lending prospective support to the hypothesis that more restrained adolescents save calories in the evening. These results have potential implications for long term health, since timing and distribution of energy intake over the day are discussed as risk factors for the development of obesity in children [65, 66]. To our knowledge up to now only one study with a small sample (n = 143) of female adults with overweight and obesity has examined the association between RE and the distribution of energy intake over the day [67]. In line with our findings, RE correlated positively with the proportion of energy intake consumed earlier in the day and inversely with the proportion of energy intake consumed after 5:00 pm [67]. Therefore, based on our results, we propose that also adolescents and especially girls with higher levels of RE show a shift in energy intake towards higher morning energy intake and lower evening energy intake. Breakfast is often declared as the most important meal of the day and in public perception regular breakfast intake is considered as part of a healthy lifestyle and prevents weight gain [68–71]. Further, reducing energy intake in the evening is a popular recommendation in various diets. Since RE represents a cognitive weight control behavior, it seems plausible, that adults and adolescents with higher levels of RE consciously adhere to public dietary advices promoting health and weight loss. Additionally, in some other studies, consuming a higher proportion of energy intake in the morning has been associated with a lower total energy intake [18, 72], like it has been seen in adolescents with higher levels of RE in the present sample.

Results reported by de Castro et al. suggest that higher morning energy intake is associated with lower total energy intake because morning energy intake is particularly satiating [18]. Although RE is marked by cognitive control and therefore seems to be largely independent of feelings of hunger and satiety, restrained eaters might consciously consume higher morning energy intake due to the promoted health effects and the long-lasting satiating effect which facilitates the restriction of energy intake over the course of the day and results not only in a shift in energy intake compared to non-restrained peers but also in a lower total energy intake. Our results further suggest that restrained adolescents restrict their caloric intake by consuming less energy in the evening, which is also supported by the above mentioned findings in adults of Leblanc et al. [67]. Thus, restrained adolescents would consciously restrict their energy intake at a time of the day when intake is supposed to promote weight gain and, according to de Castro et al., appears to be less satiating [17, 18], which makes people more vulnerable to overeating late in the day. In concordance with this, there is evidence that people can learn to better self-regulate by practicing and that people who successfully control their eating show improved self-regulation [5, 73]. Regular breakfast, higher energy intake in the morning and lower energy intake in the evening have often been associated with lower measures of obesity and a number of health benefits [66, 71, 74–76]. Accordingly, the successful practice of self-regulation by restrained adolescents in the present sample seems to contribute to a lifestyle with a lower risk for the development of obesity. At first glance, this seems contradictory to the fact that, similar to other studies [27–30], adolescents with higher levels of RE showed higher BMI in the present sample. However, it has been shown before that higher BMI and higher body weight are associated with a more frequent reporting of restrained eating in adolescents [26, 77, 78] and according to Snoek et al., positive associations between RE and BMI in adolescents should mainly be interpreted in the sense that higher BMI predicts greater RE [77]. Nevertheless, there is also longitudinal evidence supporting the opposite pathway [79, 80]. In accordance with our findings, RE has been shown to be beneficial for fostering a moderate eating behavior and for effective weight control in adults with overweight [5, 81]. Indeed, dieting in adolescence has also been related to a more effective complying with health messages, but healthy eating can also be used to hide risky weight reduction behaviors [14]. In concordance with that, RE has been linked to eating disorders and under- and malnutrition, which may have serious consequences in adolescence [5, 45, 46, 82]. Further, morning energy intake and evening energy intake together contribute to approximately 50–60% of total energy intake only, which means that restrained eaters could additionally restrict their caloric intake at another time of the day e. g. at lunch, as also found by Leblanc et al. [67]. Therefore, caution is needed in generalizing the present results.

A study by Schubert and Randler [83] found a positive association between morningness (i.e. preference for an early sleep timing) and RE. Morningness, in turn, has also been shown to be associated with a higher energy intake at breakfast, less breakfast skipping and lower energy intake in the evening compared to eveningness [84–87]. Thus, the positive association between RE and morning energy intake may be explained by the fact that restrained eaters are more likely to be early chronotypes. However no causality is given for this association. Therefore, one could speculate, that the cognitive self-control of restrained eaters is even reflected in their sleeping behavior in a way that restrained eaters might consciously go to bed and get up early because in public perception this behavior is often considered as part of a healthy lifestyle. This would lead to the question, whether the cognitive self-control inhibits the adaption of the eating- and sleep-behavior to the individual chronotype, which might have negative health effects in the form of metabolic disorders [88–91]. However, this theory demands further examination.

### Strengths and limitations

The strengths of this study are the prospective design of the DONALD study with a long follow-up and repeated measurements on the same individual. RE and food intake of participants are repeatedly measured from childhood to adulthood. Therefore, changes in the investigated variables during adolescence can be determined. Repeated measurements of individuals and family relationships are taken into account in statistical analyses.

Dietary assessment was constantly measured with the same methodology (3-day weighed dietary records) and the food composition database is updated continuously. Three-day weighed dietary records are known to be the gold-standard of dietary assessment and provide detailed daytime specific information on dietary intake which allowed a definition of eating occasion frequency and snack frequency based on criteria independent of participants’ individual perceptions. Further, we used standardized consistent and well established [39, 40, 42] criteria for the definition of eating occasions, snacks, morning energy intake and evening energy intake, which makes our findings comparable over time [39] and across analyses [39, 40, 42] within the DONALD study. Concerning differences in eating occasion frequency, it might however be questionable, if three days of recording are sufficient to measure habitual eating occasion frequency [92].

RE was assessed by use of the DEBQ-K by Franzen and Florin [33], which is a reliable validated instrument for the assessment of RE in German-speaking children and adolescents [33, 36]. Satisfying values for inner consistency (Cronbach alphas: 0.91–0.94) and test-retest-reliability (r = 0.71–0.88) allow the use for follow-up measurements and measurements of change [37]. Additionally, an earlier evaluation of this questionnaire in the DONALD study showed that 90% of the adolescents judged the questionnaire as easy to understand and time expenditure was classified as appropriate [46].

One major limitation of the present study is the small sample size. However, participants who were excluded from the analyses had similar characteristics with regard to sex, age, BMI-SDS, breast-feeding, maternal overweight and maternal educational status compared to included participants, maternal employment was however lower among excluded ones (data in S3 Table). Further limitations are the lacking reliance on self-reported data on dietary intake and RE and the overall high socioeconomic background of DONALD study participants. Compared to the general German population, families with a high socioeconomic background are overrepresented in the DONALD study, which is reflected by the high maternal educational level and can be explained by the high burden of a longitudinal study. Furthermore, in comparison to representative data on the German public, proportion of overweight in participants and their mothers was low [93, 94]. For these reasons, the sample is not representative, which limits the generalizability of our results. As there are findings suggesting that socioeconomic disadvantages in early and late childhood are prospectively associated with RE in preadolescence [20], potential associations might therefore be underestimated in the present study [95]. In addition, levels of RE were rather low in the present study compared to other examinations, where the DEBQ-K was used to assess dietary restraint in healthy children [36, 60], and our observed changes in RE during adolescence can also be rated as low (Highest possible change: ± 30; change in median of RE-score [25^th^; 75^th^ percentile] for decreasing RE: -5 [-3; -9]; change in median of RE-score [25^th^; 75^th^ percentile] for increasing RE: +4 [+3; +7]).

## Conclusion

The present study provides preliminary evidence for a relevance of RE for characteristics of circadian eating pattern in a convenience sample of adolescents from the DONALD study. Our results suggest, that adolescents with higher levels of RE differ from less or non-restrained adolescents in their characteristics of circadian eating pattern. In particular, RE seems to be relevant for the distribution of daily energy intake, in a way that especially female adolescents with higher levels of RE show a shift in energy intake towards higher morning energy intake and lower evening energy intake. Furthermore, changes in RE during adolescence seem to be accompanied by changes in some characteristics of circadian eating pattern, like eating occasion frequency and evening energy intake. Therefore, RE should be considered a relevant determinant of circadian eating patterns. Further studies are needed to elucidate whether there is an interaction with chronotype.

## Supporting information

S1 Table Participant’s characteristics and dietary characteristics at baseline and at endpoint stratified for boys (n = 101) and girls (n = 108). (PDF)

S2 Table Participant’s characteristics and dietary characteristics of the total study sample (n = 209). (PDF)

S3 Table Characteristics of excluded participants (n = 285 participants) due to missing values in the restrained questionnaire, lack of repeated restrained questionnaire, missing concurrent collected dietary record, missing data to calculate age at take-off. (PDF)

## References

[1] Garaulet M, Madrid JA. Chronobiological aspects of nutrition, metabolic syndrome and obesity. Adv Drug Deliv Rev. 2010;62(9–10):967–78. doi: 10.1016/j.addr.2010.05.005 20580916 10.1016/j.addr.2010.05.005

[2] Watanabe Y, Saito I, Henmi I, Yoshimura K, Maruyama K, Yamauchi K, et al Skipping Breakfast is Correlated with Obesity. J Rural Med. 2014;9(2):51–8. doi: 10.2185/jrm.2887 25648986 10.2185/jrm.2887 PMC4310153

[3] Oosterman JE, Kalsbeek A, la Fleur SE, Belsham DD. Impact of nutrients on circadian rhythmicity. Am J Physiol Regul Integr Comp Physiol. 2015;308(5):R337–50. doi: 10.1152/ajpregu.00322.2014 . 25519730 10.1152/ajpregu.00322.2014 PMC4346762

[4] Pietrowsky R, Straub K, Hachl P. Body dissatisfaction in female restrained eaters depends on food deprivation. Appetite. 2003;40(3):285–90. doi: 10.1016/s0195-6663(03)00012-6 12798786 10.1016/s0195-6663(03)00012-6

[5] Johnson F, Pratt M, Wardle J. Dietary restraint and self-regulation in eating behavior. Int J Obes. 2012;36(5):665–74. doi: 10.1038/ijo.2011.156 21829162 10.1038/ijo.2011.156

[6] Shunk JA, Birch LL. Validity of dietary restraint among 5- to 9-year old girls. Appetite. 2004;42(3):241–7. doi: 10.1016/j.appet.2003.11.007 15183914 10.1016/j.appet.2003.11.007

[7] Wardle J, Beales S. Restraint and food intake: an experimental study of eating patterns in the laboratory and in normal life. Behav Res Ther. 1987;25(3):179–85. 3619851 10.1016/0005-7967(87)90044-1

[8] de Castro JM. The relationship of cognitive restraint to the spontaneous food and fluid intake of free-living humans. Physiol Behav. 1995;57(2):287–95. 7716205 10.1016/0031-9384(94)00229-x

[9] Anschutz DJ, van Strien T, Van De Ven MOM, Engels RCME. Eating styles and energy intake in young women. Appetite. 2009;53(1):119–22. doi: 10.1016/j.appet.2009.03.016 19481836 10.1016/j.appet.2009.03.016

[10] Olea Lopez AL, Johnson L. Associations between restrained eating and the size and frequency of overall intake, meal, snack and drink occasions in the UK Adult National Diet and Nutrition Survey. PLoS One. 2016;11(5):e0156320 doi: 10.1371/journal.pone.0156320 27227409 10.1371/journal.pone.0156320 PMC4882017

[11] Herman CP, Mack D. Restrained and unrestrained eating. J Pers. 1975;43(4):647–60. 1206453 10.1111/j.1467-6494.1975.tb00727.x

[12] Stroebe W. Übergewicht als Schicksal? Die kognitive Steuerung des Eßverhaltens. Psychol Rundsch. 2002;53(1):14–22.

[13] Tuschl RJ, Laessle RG, Platte P, Pirke KM. Differences in food-choice frequencies between restrained and unrestrained eaters. Appetite. 1990;14(1):9–13. 2310178 10.1016/0195-6663(90)90050-i

[14] Lattimore PJ, Halford JCG. Adolescence and the diet-dieting disparity: healthy food choice or risky health behaviour? Br J Health Psychol. 2003;8(Pt 4):451–63. doi: 10.1348/135910703770238301 14614792 10.1348/135910703770238301

[15] Edmunds H, Hill AJ. Dieting and the family context of eating in young adolescent children. Int J Eat Disord. 1999;25(4):435–40. 10202654 10.1002/(sici)1098-108x(199905)25:4<435::aid-eat8>3.0.co;2-3

[16] Timlin MT, Pereira MA, Story M, Neumark-Sztainer D. Breakfast eating and weight change in a 5-year prospective analysis of adolescents: Project EAT (Eating Among Teens). Pediatrics. 2008;121(3):e638–45. doi: 10.1542/peds.2007-1035 18310183 10.1542/peds.2007-1035

[17] Boland WA, Connell PM, Vallen B. Time of day effects on the regulation of food consumption after activation of health goals. Appetite. 2013;70:47–52. doi: 10.1016/j.appet.2013.06.085 . 23816756 10.1016/j.appet.2013.06.085

[18] de Castro JM. The time of day of food intake influences overall intake in humans. J Nutr. 2004;134(1):104–11. doi: 10.1093/jn/134.1.104 14704301 10.1093/jn/134.1.104

[19] Hölling H, Schlack R. [Eating disorders in children and adolescents. First results of the German Health Interview and Examination Survey for Children and Adolescents (KiGGS)]. Bundesgesundheitsblatt Gesundheitsforschung Gesundheitsschutz. 2007;50(5–6):794–9. doi: 10.1007/s00103-007-0242-6 . 17514465 10.1007/s00103-007-0242-6

[20] Munkholm A, Olsen EM, Rask CU, Clemmensen L, Rimvall MK, Jeppesen P, et al Eating behaviours in preadolescence are associated with body dissatisfaction and mental disorders—Results of the CCC2000 study. Appetite. 2016;101:46–54. doi: 10.1016/j.appet.2016.02.020 26896837 10.1016/j.appet.2016.02.020

[21] Hölling H, Schlack R. Eating disorders in children and adolescents. First results of the German Health Interview and Examination Survey for Children and Adolescents (KiGGS). Bundesgesundhbl Gesundheitsforsch Gesundheitsschutz. 2007;50(5–6):794–9. doi: 10.1007/s00103-007-0242-6 17514465 10.1007/s00103-007-0242-6

[22] Wardle J, Beales S. Restraint, body image and food attitudes in children from 12 to 18 years. Appetite. 1986;7(3):209–17. 3800362 10.1016/s0195-6663(86)80026-5

[23] Buyken AE, Mitchell P, Ceriello A, Brand-Miller J. Optimal dietary approaches for prevention of type 2 diabetes: a life-course perspective. Diabetologia. 2010;53(3):406–18. doi: 10.1007/s00125-009-1629-8 20049415 10.1007/s00125-009-1629-8

[24] The NS, Suchindran C, North KE, Popkin BM, Gordon-Larsen P. Association of adolescent obesity with risk of severe obesity in adulthood. JAMA. 2010;304(18). doi: 10.1001/jama.2010.1635 21063014 10.1001/jama.2010.1635 PMC3076068

[25] Lee H, Lee D, Guo G, Harris KM. Trends in body mass index in adolescence and young adulthood in the United States: 1959–2002. J Adolesc Health. 2011;49(6):601–8. doi: 10.1016/j.jadohealth.2011.04.019 22098770 10.1016/j.jadohealth.2011.04.019 PMC3228354

[26] Braet C, Wydhooge K. Dietary restraint in normal weight and overweight children. A cross-sectional study. Int J Obes Relat Metab Disord. 2000;24(3):314–8. 10757624 10.1038/sj.ijo.0801129

[27] Shunk JA, Birch LL. Girls at risk for overweight at age 5 are at risk for dietary restraint, disinhibited overeating, weight concerns, and greater weight gain from 5 to 9 years. J Am Diet Assoc. 2004;104(7):1120–6. doi: 10.1016/j.jada.2004.04.031 15215771 10.1016/j.jada.2004.04.031 PMC2562311

[28] Snoek HM, Engels RC, van Strien T, Otten R. Emotional, external and restrained eating behaviour and BMI trajectories in adolescence. Appetite. 2013;67:81–7. doi: 10.1016/j.appet.2013.03.014 . 23571047 10.1016/j.appet.2013.03.014

[29] Price M, Higgs S, Lee M. Self-reported eating traits: Underlying components of food responsivity and dietary restriction are positively related to BMI. Appetite. 2015;95:203–10. Epub 2015/07/15. doi: 10.1016/j.appet.2015.07.006 . 26162952 10.1016/j.appet.2015.07.006

[30] Bisset S, Gauvin L, Potvin L, Paradis G. Association of body mass index and dietary restraint with changes in eating behaviour throughout late childhood and early adolescence: a 5-year study. Public Health Nutr. 2007;10(8):780–9. Epub 2007/03/27. doi: 10.1017/S1368980007249626 . 17381909 10.1017/S1368980007249626

[31] Kroke A, Manz F, Kersting M, Remer T, Sichert-Hellert W, Alexy U, et al The DONALD Study. History, current status and future perspectives. Eur J Nutr. 2004;43(1):45–54. doi: 10.1007/s00394-004-0445-7 14991269 10.1007/s00394-004-0445-7

[32] Buyken AE, Karaolis-Danckert N, Remer T. Association of prepubertal body composition in healthy girls and boys with the timing of early and late pubertal markers. Am J Clin Nutr. 2009;89(1):221–30. doi: 10.3945/ajcn.2008.26733 . 19056586 10.3945/ajcn.2008.26733

[33] Franzen S, Florin I. Der Dutch Eating Behavior Questionnaire für Kinder (DEBQ-K)—Ein Fragebogen zur Erfassung gezügelten Essverhaltens. Kindheit und Entwicklung. 1997;6:116–22.

[34] Grunert SC. Ein Inventar zur Erfassung von Selbstaussagen zum Ernährungsverhalten. Diagnostica. 1989;35(2):167–79.

[35] van Strien T, Frijters JER, Bergers GPA, Defares PB. The Dutch Eating Behavior Questionnaire (DEBQ) for assessment of restrained, emotional, and external eating behavior. Int J Eat Disord. 1986;5(2):295–315. doi: 10.1002/1098-108x(198602)5:2<295::aid-eat2260050209>3.0.co;2-t

[36] Dannigkeit N, Köster G, Tuschen-Caffier B. Ist primäre Prävention von Essstörungen langfristig wirksam? Ergebnisse zur Evaluation eines Trainingsprogramms an Schulen. Zeitschrift für Gesundheitspsychologie. 2005;13(2):79–91.

[37] Barkmann C, Schulte-Markwort M, Brähler E. Klinisch-psychiatrische Ratingskalen für das Kindes- und Jugendalter Göttingen: Hogrefe; 2011.

[38] Sichert-Hellert W, Kersting M, Chahda C, Schäfer R, Kroke A. German food composition database for dietary evaluations in children and adolescents. J Food Compost Anal. 2007;20:63–70.

[39] Rossbach S, Diederichs T, Bolzenius K, Herder C, Buyken AE, Alexy U. Age and time trends in eating frequency and duration of nightly fasting of German children and adolescents. Eur J Nutr. 2016 doi: 10.1007/s00394-016-1286-x 27492443 10.1007/s00394-016-1286-x

[40] Buyken AE, Trauner K, Gunther AL, Kroke A, Remer T. Breakfast glycemic index affects subsequent daily energy intake in free-living healthy children. Am J Clin Nutr. 2007;86(4):980–7. doi: 10.1093/ajcn/86.4.980 . 17921374 10.1093/ajcn/86.4.980

[41] Leech RM, Worsley A, Timperio A, McNaughton SA. Understanding meal patterns: definitions, methodology and impact on nutrient intake and diet quality. Nutr Res Rev. 2015;28(1):1–21. doi: 10.1017/S0954422414000262 25790334 10.1017/S0954422414000262 PMC4501369

[42] Diederichs T, Rossbach S, Herder C, Alexy U, Buyken AE. Relevance of morning and evening energy and macronutrient intake during childhood for body composition in early adolescence. Nutrients. 2016;8(11). Epub 2016/11/12. doi: 10.3390/nu8110716 27834901 10.3390/nu8110716 PMC5133102

[43] Kromeyer-Hauschild K, Wabitsch M, Kunze D, Gellert F, Geiß HC, Hesse V, et al Perzentile für den Body-mass-Index für das Kindes- und Jugendalter unter Heranziehung verschiedener deutscher Stichproben. Monatsschrift für Kinderheilkunde. 2001;(149):807–18.

[44] Story M, Neumark-Sztainer D, French S. Individual and environmental influences on adolescent eating behaviors. J Am Diet Assoc. 2002;102(3 Suppl):S40–51. . 11902388 10.1016/s0002-8223(02)90421-9

[45] Rolland-Cachera MF, Bellisle F, Deheeger M. Nutritional status and food intake in adolescents living in Western Europe. Eur J Clin Nutr. 2000;54 Suppl 1:S41–6. 10805037 10.1038/sj.ejcn.1600983

[46] Günther ALB. Gezügeltes Essverhalten und Einstellungen zu Ernährung und Gewicht bei Jugendlichen: Ergebnisse der DONALD Studie. EU. 2007:456–62.

[47] Roth M. Prädiktoren gezügelten Essverhaltens bei Jugendlichen. Zeitschrift für medizinische Psychologie. 1998;(4):158–62.

[48] Birch L, Savage JS, Ventura A. Influences on the Development of Children’s Eating Behaviours: From Infancy to Adolescence. Can J Diet Pract Res. 2007;68(1):s1–s56. Epub 2007/01/01. 19430591 PMC2678872

[49] Disantis KI, Collins BN, Fisher JO, Davey A. Do infants fed directly from the breast have improved appetite regulation and slower growth during early childhood compared with infants fed from a bottle? Int J Behav Nutr Phys Act. 2011;8:89 doi: 10.1186/1479-5868-8-89 21849028 10.1186/1479-5868-8-89 PMC3170240

[50] Li R, Fein SB, Grummer-Strawn LM. Do infants fed from bottles lack self-regulation of milk intake compared with directly breastfed infants? Pediatrics. 2010;125(6):e1386–93. doi: 10.1542/peds.2009-2549 . 20457676 10.1542/peds.2009-2549

[51] Snoek HM, van Strien T, Janssens JM, Engels RC. Longitudinal relationships between fathers', mothers', and adolescents’ restrained eating. Appetite. 2009;52(2):461–8. doi: 10.1016/j.appet.2008.12.009 . 19135489 10.1016/j.appet.2008.12.009

[52] Francis LA, Birch LL. Maternal influences on daughters’ restrained eating behavior. Health Psychol. 2005;24(6):548–54. Epub 2005/11/17. doi: 10.1037/0278-6133.24.6.548 16287400 10.1037/0278-6133.24.6.548 PMC2562308

[53] Carper JL, Orlet Fisher J, Birch LL. Young girls’ emerging dietary restraint and disinhibition are related to parental control in child feeding. Appetite. 2000;35(2):121–9. doi: 10.1006/appe.2000.0343 10986105 10.1006/appe.2000.0343

[54] Fernandez-Alvira JM, Bornhorst C, Bammann K, Gwozdz W, Krogh V, Hebestreit A, et al Prospective associations between socio-economic status and dietary patterns in European children: the Identification and Prevention of Dietary- and Lifestyle-induced Health Effects in Children and Infants (IDEFICS) Study. Br J Nutr. 2015;113(3):517–25. doi: 10.1017/S0007114514003663 25563904 10.1017/S0007114514003663

[55] Munkholm A, Olsen EM, Rask CU, Clemmensen L, Rimvall MK, Jeppesen P, et al Early Predictors of Eating Problems in Preadolescence-A Prospective Birth Cohort Study. J Adolesc Health. 2016;58(5):533–42. Epub 2016/04/25. doi: 10.1016/j.jadohealth.2016.01.006 . 27107908 10.1016/j.jadohealth.2016.01.006

[56] Maldonado G, Greenland S. Simulation study of confounder-selection strategies. Am J Epidemiol. 1993;138(11):923–36. . 8256780 10.1093/oxfordjournals.aje.a116813

[57] Kirkwood BRS, Jonathan AC. Essential Medical Statistics. 2 ed: Blackwell Science: Malde; 2003.

[58] Libuda L, Alexy U, Kersting M. Time trends in dietary fat intake in a sample of German children and adolescents between 2000 and 2010: not quantity, but quality is the issue. Br J Nutr. 2014;111(1):141–50. doi: 10.1017/S0007114513002031 . 23830595 10.1017/S0007114513002031

[59] Laessle RG, Tuschl RJ, Kotthaus BC, Pirke KM. A comparison of the validity of three scales for the assessment of dietary restraint. J Abnorm Psychol. 1989;98(4):504–7. 2592686 10.1037//0021-843x.98.4.504

[60] Tuschen-Caffier B, Pook M, Hilbert A. Diagnostik von Essstörungen und Adipositas. Göttingen: Hogrefe; 2005.

[61] Asbeck I, Mast M, Bierwag A, Westenhofer J, Acheson KJ, Muller MJ. Severe underreporting of energy intake in normal weight subjects: use of an appropriate standard and relation to restrained eating. Public Health Nutr. 2002;5(5):683–90. Epub 2002/10/10. doi: 10.1079/PHN2002337 . 12372163 10.1079/PHN2002337

[62] Babio N, Canals J, Fernandez-Ballart J, Arija V. Non-clinical adolescent girls at risk of eating disorder: under-reporters or restrained eaters? Nutr Hosp. 2008;23(1):27–34. . 18372942

[63] Leech RM, Worsley A, Timperio A, McNaughton SA. Characterizing eating patterns: a comparison of eating occasion definitions. Am J Clin Nutr. 2015;102(5):1229–37. doi: 10.3945/ajcn.115.114660 . 26447152 10.3945/ajcn.115.114660

[64] Heatherton TF, Herman CP, Polivy J, King GA, McGree ST. The (mis)measurement of restraint: An analysis of conceptual and psychometric issues. J Abnorm Psychol. 1988;97(1):19–28. doi: 10.1037/0021-843x.97.1.19 3280636 10.1037//0021-843x.97.1.19

[65] Maffeis C, Provera S, Filippi L, Sidoti G, Schena S, Pinelli L, et al Distribution of food intake as a risk factor for childhood obesity. Int J Obes Relat Metab Disord. 2000;24(1):75–80. 10702754 10.1038/sj.ijo.0801088

[66] Eng S, Wagstaff DA, Kranz S. Eating late in the evening is associated with childhood obesity in some age groups but not in all children: The relationship between time of consumption and body weight status in U.S. children. Int J Behav Nutr Phys Act. 2009;6(1):27 doi: 10.1186/1479-5868-6-27 19460145 10.1186/1479-5868-6-27 PMC2689163

[67] Leblanc V, Provencher V, Begin C, Gagnon-Girouard M-P, Corneau L, Tremblay A, et al Associations between eating patterns, dietary intakes and eating behaviors in premenopausal overweight women. Eat Behav. 2012;13(2):162–5. doi: 10.1016/j.eatbeh.2011.12.002 22365804 10.1016/j.eatbeh.2011.12.002

[68] Betts JA, Richardson JD, Chowdhury EA, Holman GD, Tsintzas K, Thompson D. The causal role of breakfast in energy balance and health: a randomized controlled trial in lean adults. Am J Clin Nutr. 2014;100(2):539–47. Epub 2014/06/06. doi: 10.3945/ajcn.114.083402 24898233 10.3945/ajcn.114.083402 PMC4095658

[69] Chowdhury EA, Richardson JD, Holman GD, Tsintzas K, Thompson D, Betts JA. The causal role of breakfast in energy balance and health: a randomized controlled trial in obese adults. Am J Clin Nutr. 2016;103(3):747–56. Epub 2016/02/13. doi: 10.3945/ajcn.115.122044 26864365 10.3945/ajcn.115.122044 PMC4763497

[70] Betts JA, Chowdhury EA, Gonzalez JT, Richardson JD, Tsintzas K, Thompson D. Is breakfast the most important meal of the day? Proc Nutr Soc. 2016;75(4):464–74. Epub 2016/06/14. doi: 10.1017/S0029665116000318 . 27292940 10.1017/S0029665116000318

[71] Szajewska H, Ruszczynski M. Systematic review demonstrating that breakfast consumption influences body weight outcomes in children and adolescents in Europe. Crit Rev Food Sci Nutr. 2010;50(2):113–9. Epub 2010/01/30. doi: 10.1080/10408390903467514 . 20112153 10.1080/10408390903467514

[72] Tani Y, Asakura K, Sasaki S, Hirota N, Notsu A, Todoriki H, et al Higher proportion of total and fat energy intake during the morning may reduce absolute intake of energy within the day. An observational study in free-living Japanese adults. Appetite. 2015;92:66–73. doi: 10.1016/j.appet.2015.04.071 25937510 10.1016/j.appet.2015.04.071

[73] Kuijer R, de Ridder D, Ouwehand C, Houx B, van den Bos R. Dieting as a case of behavioural decision making. Does self-control matter? Appetite. 2008;51(3):506–11. doi: 10.1016/j.appet.2008.03.014 . 18479777 10.1016/j.appet.2008.03.014

[74] Wang JB, Patterson RE, Ang A, Emond JA, Shetty N, Arab L. Timing of energy intake during the day is associated with the risk of obesity in adults. Journal of human nutrition and dietetics: the official journal of the British Dietetic Association. 2014;27 Suppl 2:255–62. doi: 10.1111/jhn.12141 23808897 10.1111/jhn.12141

[75] Bo S, Musso G, Beccuti G, Fadda M, Fedele D, Gambino R, et al Consuming more of daily caloric intake at dinner predisposes to obesity. A 6-year population-based prospective cohort study. PLoS One. 2014;9(9):e108467 doi: 10.1371/journal.pone.0108467 25250617 10.1371/journal.pone.0108467 PMC4177396

[76] Wijtzes AI, Jansen W, Bouthoorn SH, van Lenthe FJ, Franco OH, Hofman A, et al Meal-skipping behaviors and body fat in 6-year-old children. J Pediatr. 2016;168:118–25 e2. Epub 2015/11/02. doi: 10.1016/j.jpeds.2015.09.039 . 26520914 10.1016/j.jpeds.2015.09.039

[77] Snoek HM, van Strien T, Janssens JM, Engels RC. Restrained eating and BMI: a longitudinal study among adolescents. Health Psychol. 2008;27(6):753–9. Epub 2008/11/26. doi: 10.1037/0278-6133.27.6.753 . 19025271 10.1037/0278-6133.27.6.753

[78] Braet C, Claus L, Goossens L, Moens E, Van Vlierberghe L, Soetens B. Differences in eating style between overweight and normal-weight youngsters. J Health Psychol. 2008;13(6):733–43. Epub 2008/08/14. doi: 10.1177/1359105308093850 . 18697886 10.1177/1359105308093850

[79] Neumark-Sztainer D, Wall M, Story M, Standish AR. Dieting and unhealthy weight control behaviors during adolescence: associations with 10-year changes in body mass index. J Adolesc Health. 2012;50(1):80–6. Epub 2011/12/23. doi: 10.1016/j.jadohealth.2011.05.010 . 22188838 10.1016/j.jadohealth.2011.05.010 PMC3245517

[80] Field AE, Austin SB, Taylor CB, Malspeis S, Rosner B, Rockett HR, et al Relation between dieting and weight change among preadolescents and adolescents. Pediatrics. 2003;112(4):900–6. 14523184 10.1542/peds.112.4.900

[81] Schaumberg K, Anderson DA, Anderson LM, Reilly EE, Gorrell S. Dietary restraint: what’s the harm? A review of the relationship between dietary restraint, weight trajectory and the development of eating pathology. Clin Obes. 2016;6(2):89–100. Epub 2016/02/05. doi: 10.1111/cob.12134 . 26841705 10.1111/cob.12134

[82] Herbold NH, Frates SE. Update of nutrition guidelines for the teen: trends and concerns. Curr Opin Pediatr. 2000;12(4):303–9. 10943808 10.1097/00008480-200008000-00003

[83] Schubert E, Randler C. Association between chronotype and the constructs of the Three-Factor-Eating-Questionnaire. Appetite. 2008;51(3):501–5. doi: 10.1016/j.appet.2008.03.018 18479778 10.1016/j.appet.2008.03.018

[84] Munoz JS, Canavate R, Hernandez CM, Cara-Salmeron V, Morante JJ. The association among chronotype, timing of food intake and food preferences depends on body mass status. Eur J Clin Nutr. 2016 doi: 10.1038/ejcn.2016.182 . 27650874 10.1038/ejcn.2016.182

[85] Maukonen M, Kanerva N, Partonen T, Kronholm E, Tapanainen H, Kontto J, et al Chronotype differences in timing of energy and macronutrient intakes: A population-based study in adults. Obesity (Silver Spring). 2017;25(3):608–15. doi: 10.1002/oby.21747 . 28229553 10.1002/oby.21747

[86] Reutrakul S, Hood MM, Crowley SJ, Morgan MK, Teodori M, Knutson KL. The relationship between breakfast skipping, chronotype, and glycemic control in type 2 diabetes. Chronobiol Int. 2014;31(1):64–71. doi: 10.3109/07420528.2013.821614 . 24094031 10.3109/07420528.2013.821614

[87] Lucassen EA, Zhao X, Rother KI, Mattingly MS, Courville AB, de Jonge L, et al Evening chronotype is associated with changes in eating behavior, more sleep apnea, and increased stress hormones in short sleeping obese individuals. PLoS One. 2013;8(3):e56519 Epub 2013/03/14. doi: 10.1371/journal.pone.0056519 . 23483886 10.1371/journal.pone.0056519 PMC3590198

[88] Cagampang FR, Bruce KD. The role of the circadian clock system in nutrition and metabolism. Br J Nutr. 2012;108(3):381–92. doi: 10.1017/S0007114512002139 22676899 10.1017/S0007114512002139

[89] Ekmekcioglu C, Touitou Y. Chronobiological aspects of food intake and metabolism and their relevance on energy balance and weight regulation. Obes Rev. 2011;12(1):14–25. doi: 10.1111/j.1467-789X.2010.00716.x 20122134 10.1111/j.1467-789X.2010.00716.x

[90] Roenneberg T, Kuehnle T, Juda M, Kantermann T, Allebrandt K, Gordijn M, et al Epidemiology of the human circadian clock. Sleep Med Rev. 2007;11(6):429–38. doi: 10.1016/j.smrv.2007.07.005 17936039 10.1016/j.smrv.2007.07.005

[91] Roenneberg T, Merrow M. The circadian clock and human health. Curr Biol. 2016;26(10):R432–43. Epub 2016/05/25. doi: 10.1016/j.cub.2016.04.011 . 27218855 10.1016/j.cub.2016.04.011

[92] Longnecker MP, Harper JM, Kim S. Eating frequency in the Nationwide Food Consumption Survey (U.S.A.), 1987–1988. Appetite. 1997;29(1):55–9. doi: 10.1006/appe.1997.0094 . 9268425 10.1006/appe.1997.0094

[93] Mensink GB, Schienkiewitz A, Haftenberger M, Lampert T, Ziese T, Scheidt-Nave C. [Overweight and obesity in Germany: results of the German Health Interview and Examination Survey for Adults (DEGS1)]. Bundesgesundheitsblatt Gesundheitsforschung Gesundheitsschutz. 2013;56(5–6):786–94. doi: 10.1007/s00103-012-1656-3 . 23703499 10.1007/s00103-012-1656-3

[94] Kurth BM, Schaffrath Rosario A. [The prevalence of overweight and obese children and adolescents living in Germany. Results of the German Health Interview and Examination Survey for Children and Adolescents (KiGGS)]. Bundesgesundheitsblatt Gesundheitsforschung Gesundheitsschutz. 2007;50(5–6):736–43. doi: 10.1007/s00103-007-0235-5 . 17514458 10.1007/s00103-007-0235-5

[95] Buyken AE, Alexy U, Kersting M, Remer T. [The DONALD cohort. An updated overview on 25 years of research based on the Dortmund Nutritional and Anthropometric Longitudinally Designed study]. Bundesgesundheitsblatt Gesundheitsforschung Gesundheitsschutz. 2012;55(6–7):875–84. doi: 10.1007/s00103-012-1503-6 . 22736170 10.1007/s00103-012-1503-6

