# Supplementary material for: The relevance of restrained eating behavior for circadian eating patterns in adolescents
Source: PLoS One. 2018 May 23;13(5):e0197131. doi: 10.1371/journal.pone.0197131 (PMC5965828; doi:10.1371/journal.pone.0197131)
Supplement: S1 Table — (PDF) [file pone.0197131.s001.pdf]

S1 Table: Participant's characteristics and dietary characteristics at baseline and at endpoint stratified for boys (n=101) and girls (n=108)

	Baseline		Endpoint	
	♀	♂	♀	♂
**Participant's characteristics**				
Age [years]				
Median (25th; 75th percentile)	11 (11; 14)	11 (11; 14)	18 (14; 18)	18 (14; 18)
Mean (SD)	12.4 (1.8)	12.6 (1.9)	16.4 (2.0)	16.7 (2.0)
RE Score [0-30]	6 (1; 11)	5 (1; 11)	6 (2; 13)	4 (1; 9)
Minimum	0	0	0	0
Maximum	24	20	24	18
BMI-SDS	0.02 (-0.58; 0.62)	0.18 (-0.68; 0.77)	0.26 (-0.41; 0.93)	0.32 (-0.68; 1.02)
Body weight status [a]				
Normal weight [n (%)]	93 (86)	79 (78)	85 (79)	72 (71)
Underweight [n (%)]	8 (7)	8 (8)	11 (10)	11 (11)
Overweight [n (%)]	4 (4)	10 (10)	7 (6)	12 (12)
Obesity [n (%)]	3 (3)	4 (4)	5 (5)	6 (6)
**Parental characteristics**				
Maternal overweight [b] [n (%)]	46 (43)	37 (37)	48 (44)	39 (39)
High maternal educational status [c] [n (%)]	80 (74)	68 (67)	81 (75)	70 (69)
Maternal employment [n (%)]	85 (79)	77 (76)	85 (79)	82 (81)
**Dietary characteristics**				
Number of recorded weekdays				
1 [n (%)]	42 (39)	41 (41)	32 (30)	34 (34)
2 [n (%)]	23 (21)	20 (20)	19 (18)	23 (23)
3 [n (%)]	43 (40)	40 (40)	57 (53)	44 (44)
Total energy intake [kcal]	1712 (1478; 1939)	2016 (1793; 2448)	1768 (1487; 2057)	2349 (2014; 2773)
Morning energy intake [%E]	27.7 (22.0; 32.9)	24.5 (19.4; 31.7)	24.8 (16.6; 30.5)	22.1 (13.5; 28.3)
No energy intake in the morning				
Never [n (%)]	98 (91)	85 (84)	75 (69)	68 (67)
On 1 day [n (%)]	9 (8)	11 (11)	21 (19)	18 (18)
On 2 days [n (%)]	1 (1)	3 (3)	9 (8)	10 (10)
On 3 days [n (%)]	-	2 (2)	3 (3)	5 (5)
Evening energy intake [%E]	27.0 (21.4; 34.2)	30.6 (24.9; 35.6)	29.9 (21.0 36.5)	34.5 (27.8; 40.1)
No energy intake in the evening				
Never [n (%)]	92 (85)	90 (89)	90 (83)	96 (95)
On 1 day [n (%)]	14 (13)	11 (11)	10 (9)	5 (5)
On 2 days [n (%)]	2 (2)	-	8 (7)	-
On 3 days [n (%)]	-	-	-	-
Eating occasion frequency [n/day]	5.3 (4.7; 6.0)	5.3 (4.7; 6.3)	5.0 (4.3; 5.7)	5.3 (4.7; 6.0)
Snack frequency [n/day]	1.3 (0.7; 2.0)	1.3 (0.7; 2.0)	1.2 (0.7; 2.0)	1.7 (1.0; 2.3)

Unless otherwise indicated, presented values are medians (25th; 75th percentile) or frequencies (%)
Abbreviations: ATO ≙ Age at Take-Off, BMI ≙ Body Mass Index, SDS ≙ Standard Deviation Score, %E ≙ percentage of total energy intake
[a] Overweight: >90th percentile/Obesity: >97th percentile/Underweight: <10th percentile of BMI-SDS based on German reference curves [41], [b]BMI≥25, [c] ≥12 years of schooling
