# Supplementary material for: The relevance of restrained eating behavior for circadian eating patterns in adolescents
Source: PLoS One. 2018 May 23;13(5):e0197131. doi: 10.1371/journal.pone.0197131 (PMC5965828; doi:10.1371/journal.pone.0197131)
Supplement: S2 Table — (PDF) [file pone.0197131.s002.pdf]

S2 Table: Participant's characteristics and dietary characteristics of the total study sample (n=209)

**Participant's characteristics**	
n (questionnaires/dietary records)	418
n (participants)	209
Boys [n (%)]	101 (48)
Age [years]	14 (11; 18)
RE Score [0-30]	5 (1; 11)
Minimum	0
Maximum	24
ATO [years]	10 (9; 11)
BMI-SDS	0.19 (-0.78; 0.82)
Body weight status [a]	
Normal weight [n (%)]	329 (79)
Underweight [n (%)]	38 (9)
Overweight [n (%)]	33 (8)
Obesity [n (%)]	18 (4)
Breast-fed ≥ 4 months [n (%)]	282 (67)
**Parental characteristics**	
Maternal overweight [b] [n (%)]	170 (41)
High maternal educational status [c] [n (%)]	299 (72)
Maternal employment [n (%)]	329 (79)
**Dietary characteristics**	
Number of recorded weekdays	
1 [n (%)]	149 (36)
2 [n (%)]	85 (20)
3 [n (%)]	184 (44)
Total energy intake [kcal]	1935 (1608; 2276)
Morning energy intake [%E]	24.5 (18.1; 31.4)
No energy intake in the morning	
Never [n (%)]	326 (78)
On 1 day [n (%)]	59 (14)
On 2 days [n (%)]	23 (6)
On 3 days [n (%)]	10 (2)
Evening energy intake [%E]	30.1 (24.0; 36.7)
No energy intake in the evening	
Never [n (%)]	368 (88 %)
On 1 day [n (%)]	40 (10 %)
On 2 days [n (%)]	10 (2 %)
On 3 days [n (%)]	-
Eating occasion frequency [n/day]	5.3 (4.7; 6.0)
Snack frequency [n/day]	1.3 (0.7; 2.0)

Presented values are medians (25th; 75th percentile) or frequencies (%)
Abbreviations: ATO ≙ Age at Take-Off, BMI ≙ Body Mass Index, SDS ≙ Standard Deviation Score, %E ≙ percentage of total energy intake
[a] Overweight: >90th percentile/Obesity: >97th percentile/Underweight: <10th percentile of BMI-SDS based on German reference curves [41], [b] BMI ≥25, [c] ≥12 years of schooling
