# Supplementary material for: The relevance of restrained eating behavior for circadian eating patterns in adolescents
Source: PLoS One. 2018 May 23;13(5):e0197131. doi: 10.1371/journal.pone.0197131 (PMC5965828; doi:10.1371/journal.pone.0197131)
Supplement: S3 Table — (PDF) [file pone.0197131.s003.pdf]

S3 Table: Characteristics of excluded participants (n=285 participants) due to missing values in the restrained questionnaire, lack of repeated restrained questionnaire, missing concurrent collected dietary record, missing data to calculate age at take-off

**Participant's characteristics**	
n (questionnaires/dietary records)	386
n (participants)	285
Boys [n (%)]	218 (56)
Age [years]	14 (11; 15)
BMI-SDS	0.08 (-0.63; 0.70)
Body weight status [a]	
Normal weight [n (%)]	316 (82)
Underweight [n (%)]	28 (7)
Overweight [n (%)]	29 (8)
Obesity [n (%)]	13 (3)
Breast-fed ≥ 4 months [b] [n (%)]	238 (67)
**Parental characteristics**	
Maternal overweight [c] [n (%)]	102 (26)
High maternal educational status [d] [n (%)]	273 (71)
Maternal employment [n (%)]	73 (19)

Presented values are medians (25th; 75th percentile) or frequencies (%)
Abbreviations: ATO ≙ Age at Take-Off, BMI ≙ Body Mass Index, SDS ≙ Standard Deviation Score
[a] Overweight: >90th percentile/Obesity: >97th percentile/Underweight: <10th percentile of BMI-SDS based on German reference curves [43], [b] including 29 missing values for 19 participants, [c] BMI ≥25, [d] ≥12 years of schooling
